# Low-Asymmetry Interface for Multiuser VR Experiences with Both HMD and Non-HMD Users

**DOI:** 10.3390/s21020397

**Published:** 2021-01-08

**Authors:** Qimeng Zhang, Ji-Su Ban, Mingyu Kim, Hae Won Byun, Chang-Hun Kim

**Affiliations:** 1 Interdisciplinary Program in Visual Information Processing, Korea University, Seoul 02841, Korea; zoe1024@korea.ac.kr (Q.Z.); vban@korea.ac.kr (J.-S.B.); kmg2917@korea.ac.kr (M.K.); 2 Department of Information Systems Engineering, Sungshin Women’s University, Seoul 02844, Korea; hyewon@sungshin.ac.kr; 3 Department of Computer Science and Engineering, Korea University, Seoul 02841, Korea

**Keywords:** low-asymmetry interface, walking simulator, shared experience, multiuser VR

## Abstract

We propose a low-asymmetry interface to improve the presence of non-head-mounted-display (non-HMD) users in shared virtual reality (VR) experiences with HMD users. The low-asymmetry interface ensures that the HMD and non-HMD users’ perception of the VR environment is almost similar. That is, the point-of-view asymmetry and behavior asymmetry between HMD and non-HMD users are reduced. Our system comprises a portable mobile device as a visual display to provide a changing PoV for the non-HMD user and a walking simulator as an in-place walking detection sensor to enable the same level of realistic and unrestricted physical-walking-based locomotion for all users. Because this allows non-HMD users to experience the same level of visualization and free movement as HMD users, both of them can engage as the main actors in movement scenarios. Our user study revealed that the low-asymmetry interface enables non-HMD users to feel a presence similar to that of the HMD users when performing equivalent locomotion tasks in a virtual environment. Furthermore, our system can enable one HMD user and multiple non-HMD users to participate together in a virtual world; moreover, our experiments show that the non-HMD user satisfaction increases with the number of non-HMD participants owing to increased presence and enjoyment.

## 1. Introduction

Improving the immersion and presence of users is the principal issue in virtual reality (VR), and several studies have explored this subject. Most of these studies have focused on improving the immersion and presence of head-mounted display (HMD) users. Recently, there have been proposals [1] to enhance the immersion and presence of non-HMD users to enable both types of users to achieve a co-located, although asymmetric, VR experience. Such proposals are driven by the different engagement levels required by various virtual environment (VE) participants. Numerous VR applications and scenarios have been employed in diverse fields such as education, health care, physical training, and home entertainment. Among these applications, in addition to the participating HMD users, the proportion of non-HMD users who experience the VR environment via a desktop PC or projector display [2] is also large and increasing. Particularly, given the popularity of social VR applications such as VR Chat [3], several users who may not wear an HMD device for certain personal reasons (such as VR sickness, unaffordable cost, inconvenient locations, or preference for a brief VR experience) may still like to participate. This makes enabling the non-HMD users to engage in a VE and improving their immersion and presence become critical issues.

Non-HMD and HMD users have asymmetric experiences, devices, and roles in immersive VR. In a previous study [4], asymmetric collaboration was divided into low, medium, and high levels of asymmetry. Among these, high-level asymmetry refers to a substantial difference in the VR experience of HMD and non-HMD users, such as in the verbal conveyance of information. In contrast, the difference between HMD and non-HMD users in low-level asymmetry can be small, such as the non-HMD users being able to change the perspective or influence the environment. Setups with various levels of asymmetry differently influence the capability of users to complete tasks in a VE, thereby affecting presence and immersion. The authors of [5] proposed an asymmetric user interface to enhance the interaction between non-HMD and HMD users and to provide non-HMD users with first- and third-person perspectives to enhance user immersion. ShareVR’s research [1] explored the effectiveness of co-located asymmetric interaction between HMD and non-HMD users and used a tracked display and floor projection to visualize the virtual space for the non-HMD users, thereby increasing their immersion and enjoyment. In these studies, because both types of users could change the perspective and influence the environment, the related setups correspond to the low-asymmetry realm. However, because the non-HMD visual display is generally a fixed desktop PC [5] or a wired tracked display [1] instead of a portable wireless device, the studies have limitations arising from the difficulties experienced by non-HMD users in engaging with the VE in any location. In addition, realistic locomotion contributes substantially to the immersion and presence in VR [6]. In existing methods, a non-HMD user navigating a VE usually employs keyboards and controller devices or their position is tracked via an external projection sensor [2,7]. These methods are more likely to cause simulation sickness and general dissatisfaction, as the body presence of non-HMD users is low and the tracking equipment can be burdensome and/or expensive. Moreover, nearly all these studies may apply well to one-to-one environments involving HMD and non-HMD users; however, they will have a limited application in one-to-many environments (one HMD user and multiple non-HMD users) because of the size of the real activity space or the restricted projection range of the projectors used.

In this paper, we propose a low-asymmetry interface to enable an increased presence of non-HMD users in a locomotion VE, so that both types of users can experience similar presence and enjoyment, and enable multiple non-HMD users to engage in the VE in any location. In the proposed system, to ensure the freedom of a non-HMD user’s viewing perspective, we provide a portable mobile device as a visual display and track the non-HMD user’s head direction via the gyro sensor of the mobile device. Inspired by [8] (whose research presented a walking simulator device for HMD users to reduce VR sickness and enabled walking realism), we provide a walking simulator for HMD users and a mobile device for non-HMD users, enabling physical movement to be translated to movement in the VE for all users. We simplified the walking simulator and algorithm proposed by [8] to reduce the burden of wearing the simulator and to measure the user’s walking motion more effectively. In addition, while using the proposed low-asymmetry interface, both HMD and non-HMD users can experience the same level of involvement in the locomotion VE because the interface can provide the same first-person viewpoint and natural walking interaction even to non-HMD users. Such users can then become the main actors, similar to HMD users, instead of playing secondary roles or acting as observers; thus, a comparable presence of the two types of users is achieved in the virtual world.

**Figure 1:**
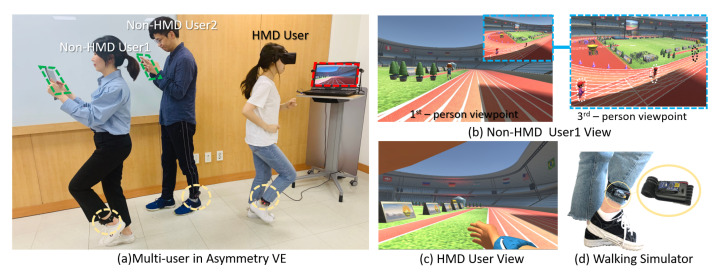
Our system enables a shared virtual reality (VR) experience with similar presence for both head-mounted display (HMD) and non-HMD users: (**a**) multiusers in the asymmetric virtual environment based on a low-asymmetry interface; (**b**) the non-HMD view and third-person viewpoint for a non-HMD user; (**c**) the HMD view; and (**d**) immersive walking interaction.

To demonstrate that our low-asymmetry interface can enable an increased presence of non-HMD users, on par with that of HMD users, and enhance the enjoyment of the non-HMD users by providing physical navigation ability to the VE participants, we performed a case study to evaluate the user experience. Our experiments involved a simple locomotion scenario with both cooperation and competition modes to prove the effectiveness of the proposed interface. Moreover, the scenario supports open participation, the hardware equipment is easy to manufacture and is wireless for non-HMD users, and the non-HMD users have no positional limitations with regard to engagement. Therefore, the proposed setup can be employed in one-to-many HMD to non-HMD user environments (as shown in Figure 1).

In summary, our contributions are as follows:

1. We propose a low-asymmetry interface for increasing the presence of non-HMD users for a shared VR experience with HMD users. By using a portable mobile device and a convenient walking simulator, non-HMD users can have their own viewpoints and realistic physical movement experiences in VE.
2. We conducted a user study that engaged both HMD and non-HMD user relationships at the same level. The performed experiments confirmed that non-HMD users feel a presence similar to that of HMD users when performing equivalent tasks in a VE.
3. Our system offers a one-to-many participation experience with one HMD and multiple non-HMD users. Our experiments show that this can satisfy non-HMD users in terms of increased enjoyment.

## 2. Related Work

### 2.1. Collaborative Activities in Asymmetric Environments

Based on research on single-user VR interfaces, scenarios entailing multi-user involvement were designed. Such environments are usually called collaborative events (CVEs). The first CVE was defined in 1998 as an interactive system for multiple users. A distributed interactive VE (DIVE) was developed as a multiuser VR system involving CVEs [9]. It is important to study multiuser scenarios that support the awareness of other participants. In [10,11], the authors discussed the manner in which people sharing a CVE are able to feel “co-presence” (awareness and understanding of the activities of other users); further, they discussed the enhancement of inter-user interactions. Several applications that use DIVE have been constructed, including VR−VIVE [12].

Novel approaches that differ from the way multiusers (VR–VR) meet and interact in a virtual space have also been suggested. These systems are designed to handle asymmetric collaborative interactions with different visualization devices and input hardware. For example, a mobile augmented reality (AR) user at an outdoor location can receive visual and aural information about tasks to be performed from an indoor user using a tablet device [13]. In a different study [14], the authors explored alternative collaborative games in which a non-VR user interacts with a VR user using an interface (a way-finding cube) that provides overview information. In [15], a commander (a non-VR user) is provided an omniscient point of view and plays an auxiliary role in helping an HMD user by providing visual information. DollhouseVR [16] entailed an asymmetric collaboration with two different views and interaction styles based on a tablet and HMD: the tablet users with a top-down view design a virtual space and the HMD users sharing the virtual space move inside this space. Oda et al. presented a system for remote assistance using an approach that enabled co-located interaction in VR and AR environments [17]. A metaphor for low-cost interactions within CVEs was presented in [18], which focused on non-immersive and semi-immersive three-dimensional (3D) interactions. By adopting a similar approach, interaction between different devices has also been presented (e.g., [19]). Here, non-HMD users primarily receive a third-person view of the virtual space information and then communicate the information to HMD users. Asymmetric PoV is commonly suggested for collaborative activities between asymmetric users. Inspired by these studies, we employed the third-person viewpoint as a reference view for non-HMD users.

Furthermore, most researchers have focused on physical interactions to enhance the HMD user experience because this enables users to feel a higher level of immersion in a VE. For instance, TurkDeck uses human actions to create an identical physical representation in a virtual space, providing realism/immersion to the HMD users via haptic feedback [20]. FaceDisplay [21] is a multiuser interaction interface with a touch display that enables non-HMD users to intervene in an HMD user’s virtual situation via touch input. However, these techniques are focused on improving the immersive experience of HMD users instead of non-HMD users. Although there have been experiments with the participation of non-HMD users, their roles were different from those of HMD users, and their methods of accessing visual information were also different, such as using a third-person view only. Their appearance in the virtual space was also different [22]. With advances in VR technology, devices are being introduced for living-room entertainment. Accordingly, asymmetric co-located VR interaction is being explored while maintaining the same level of modalities as those between HMD users and non-HMD users. In [23], an HMD user performed an experiment to collaborate with a mobile AR user to achieve a high cooperation level even in an asymmetric environment. ShareVR [1] proposed a VR system that considered non-HMD users as part of the VR experience. Within the same room, bystanders could share the VR space via a ceiling-mounted projector. They could use a controller with the display, thereby gaining visual information regarding the VE and being able to interact with the HMD user to obtain a physical experience. Despite providing individual visualization tools to non-HMD users, this system needed to be in a certain space for tracking the non-HMD users’ motion and had difficulty incorporating more participants.

In our approach, we focused on enhancing the experience of multiple non-HMD users in a VE by enabling them to experience a level of presence similar to that of HMD users while they played the same role and performed equivalent tasks. Similar to previous research, we incorporated walking interactions to increase the physical presence of non-HMD users via body movement.

### 2.2. Immersive Interfaces

Research on human–computer interaction has developed significantly in recent years. Two-dimensional generic input devices such as mice are inherently limited when used in 3D virtual applications; therefore, new input devices for 3D applications are required. Different interaction modes have been employed to develop novel technologies for increasing user immersion in a VE, including immersive interfaces that use bodily information such as haptic feedback and eye gaze [24,25]. In addition, experiments incorporating hand-motion systems have been actively conducted [26,27,28]. In one alternative approach, aural methods have also been proposed to improve immersion because the human ear can determine the locations of sound sources [29].

In [30], the authors demonstrated that when body movement is involved, engagement in the VE is increased. To improve user satisfaction with regard to the level of immersion and interaction in a virtual space, studies were performed to incorporate physical movements such that the space could be used freely (using devices such as a treadmill system). Recently, various systems that involve user movement have been developed. For example, Virtuix Omni, Cyberith Virtualizer, and Infinadeck [31,32,33] are now commercially available for nontechnical consumers. Although these devices have been able to overcome the constraints on spatial movement, they are slightly expensive. To address this issue, MAVE [8] suggested using a simple hardware system called a “walking simulator”, which reflects the footsteps of users on to a VE. Inspired by this approach, we propose using a casual interface similar to a walking simulator. Our experimental system enables users’ body movements, thereby enhancing their presence.

### 2.3. Presence

In VR, users’ presence is crucial, and it is influenced by natural movement and body information. Several studies have been conducted on the manner in which users’ body form in immersive VEs affects their cognition and attitude if it differs from their actual body form [34,35]. Matching the proprioceptive information of human body movements to the visual representations provided via computer-generated displays has been shown to enhance the immersive presence in VEs [36] and has inspired various types of research, including ours [37,38,39]. Other studies on cognition and presence have shown that humans can feel more realism in a VE [40]. Besides these, various other experiments have also been conducted for analyzing user presence. In [41], the authors analyzed the various effects of VR on human lives by considering psychological, neurological, and social phenomena. In addition, in [42], experiments were conducted on human reactions at the moment of returning from a virtual space to reality.

Based on this prior work, we designed our system to invoke a strong feeling of presence for both HMD and non-HMD users. Inspired by [30], who showed that incorporating body movement enhances such feelings, we designed our system to increase the user presence by incorporating physical movement and providing visual autonomy to non-HMD users.

## 3. Low-Asymmetry Interface

Our key idea is to increase the presence of non-HMD users by building a low-asymmetry interface that will lead to non-HMD users having a similar presence to that of HMD users. For visualization, we provided a portable mobile device to each non-HMD user to enable them to have an independent perspective and direction of movement. To incorporate movement, we provided both HMD and non-HMD users with a convenient single-leg walking simulator that ensured that the actual physical movement remained consistent with the movement in the VE. In addition, owing to the absence of immersion headsets, non-HMD users may misinterpret the walking distances in reality compared to those in the virtual space while interacting with locomotion VR content. Therefore, we provided non-HMD users with a third-person viewpoint (an overhead view) to enable them to estimate distances in the VE, thereby improving their immersion (Figure 1b). To investigate the effectiveness of the proposed low-asymmetry system, we conducted experiments with a simple application and simple rules. In this application, both HMD and non-HMD users are the main actors, enabling their virtual experiences to be compared fairly.

In the remainder of this section, we first describe immersive locomotion interactions in the low-asymmetry system (see Section 3.1). Next, we describe the viewpoint provided to non-HMD users (see Section 3.2). Finally, the implementation of the system is discussed in Section 3.3.

### 3.1. Immersive Locomotion Interaction

In a VE, users’ movements constitute fundamental VR interactions. Several tasks that require VR controllers to control user movement will cause “VR sickness”, thereby decreasing immersion. This is caused by differences among the movement frequencies of the eyes, body, and controller. Improved user immersion by detecting movement through a walking simulator or by tracking a user’s movement with a projection sensor has been demonstrated in several studies [8,43]. Out of the two approaches, directly wearing a walking simulator can allow for a wider range of movements than projector tracking, particularly when using a treadmill-style walking simulator. Because the user’s movement can be detected in-situ, there is no limitation on the size of the real physical space, enabling free movement within the VE. This has been verified experimentally [8].

**Figure 2:**
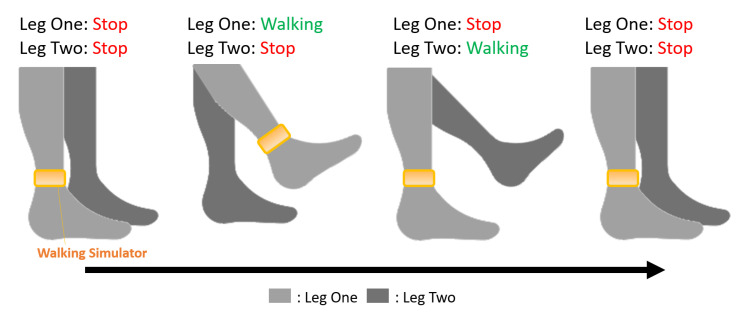
Schematic of single-leg walking decision algorithm. Images from the left to the right in the figure depict walking steps 1–4. Step 1 corresponds to the initialize state, and steps 2–4 show the state of leg 1 is decided by the walking simulator, the state of leg 2 is determined based on the previous state of leg 1.

In this work, we employed an easy-to-wear treadmill-style walking simulator similar to that proposed by [8]. However, we have simplified the walking decision algorithm from the original two-leg method to a single-leg method. This means that we only detect the movement of one leg (leg one), and the movement of the other leg (leg two) depends on the detected result of leg one and the corresponding movement (as shown in Figure 2). This is because users’ legs are usually in continuous alternating movement while walking, excluding slow-motion cases. The time difference between each leg’s stride when walking or running normally is very short (we observed the average time to be approximately 1 s), which means that single-leg-based walking estimation involves only a small error, reducing the user’s equipment burden while effectively detecting the walking movement. We prove the usability and effectiveness of the proposed single-leg-based walking decision approach in Section 4.2.

Movement detection is performed with an MPU-6050 gyro sensor, which calculates the gradient value along the x(Roll), y(Pitch), and z(Yaw) axes, and the value along the x-axis is used to judge whether walking has occurred [8]. From these measurements, we detected the axis gradient for each frame, and if the difference between the current and previous frames was greater than a specified threshold value (we used 0.5), we evaluated the movement state as true. Conversely, if the difference was less than the threshold value, the movement state was considered false. The full working of our single-leg-based in-place walking detection algorithm is shown in Algorithm 1.

In addition, while the movement state was true (while walking or running), we used the user’s calculated difference value (appropriately weighted, we used 0.01) as the speed of movement to calculate the change in distance in the VE. The user’s displacement value *D* corresponding to each frame *i* is given as

(1) $$D_{i}=F_{i}\cdot S_{i}\cdot \Delta t$$

Here, $F_{i}$ is the forward direction of the user camera, $S_{i}$ is the speed of movement, and Δt is the time step. The new position $P_{new}$ of the user in the VE is calculated as $P_{new}=P_{old}+D_{i}$.

**Algorithm 1.** Single-leg-based in-place walking detection algorithm.  1: Value ← user’s motion value (leg with walking simulator).  2: State ← user’s motion state (leg with walking simulator).  3: Roll ← roll gradient values of gyro sensor (leg with walking simulator).  4: T ← threshold for identifying movement (leg with walking simulator).  5: Value_L2 ← user’s motion value (leg without walking simulator)  6: State_L2 ← user’s motion state (leg without walking simulator)  7: **procedure**
MOTION DECISION(Value, State, Roll, T)  8:     curr_Roll ← current roll gradient value.  9:     pre_Roll ← previous roll gradient value.10:     curr_Roll = pre _ Roll11:     **if** abs (pre _Roll-curr_Roll) > T **then**12:         Value = abs (pre _Roll-curr_Roll)13:         State = True (Walking)14:     **else**15:         Value = 016:         State = False (Stop)17:     **end if**18:     pre_Value ← previous user’s motion value (leg with walking simulator)19:     pre_State ← previous user’s motion state (leg with walking simulator)20:     Value_L2 = pre_Value21:     State_L2 = pre_State22:     pre_Value = Value23:     pre_State = State24:     pre_Roll = curr_Roll25: **end procedure**

**Figure 3:**
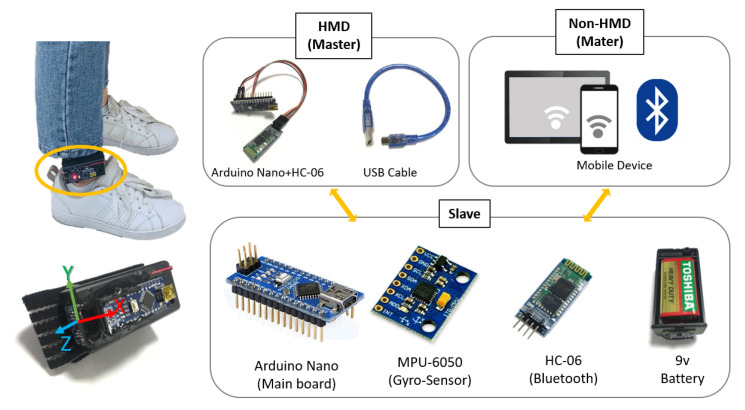
Structure of a walking simulator [8] for HMD and non-HMD users.

We used a Bluetooth module, HC-06, to transfer the data (gradient value of axis) captured by the MPU-6050 gyro sensor. Because the HMD and non-HMD users used different devices, the HMD data were received by the master device connected via a USB cable [8], whereas non-HMD users directly used Bluetooth in their portable mobile devices to receive data, as shown in Figure 3. Therefore, by employing mobile devices and walking simulators, our system enables a non-HMD user to navigate physically in the VE with freedom of movement, thereby increasing the immersion and presence experience.

### 3.2. Viewpoint for Non-HMD Users

Because non-HMD users do not wear headset devices, when walking with HMD users in a co-located environment, their field of vision will include the HMD user in the actual physical space. When moving in the VE, being presented with both the actual physical distance and the virtual distance may cause feelings of uncertainty for non-HMD users, which distracts from an immersive experience.

**Figure 4:**
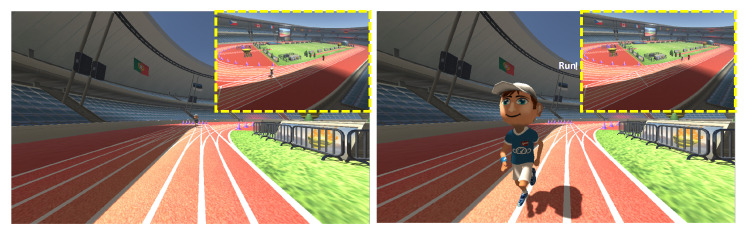
Third-person viewpoint (as shown with a yellow window) for non-HMD users. The figure shows that the HMD user(the character with the blue shirt) is facing toward the forward direction of the non-HMD user’s head. The left image shows both the users at a substantial distance. The right image shows both the users in proximity, and the text information provides hints to the non-HMD user.

To decrease this influence for non-HMD users, we provide not only a first-person viewpoint but also a third-person viewpoint for reference. This third-person viewpoint of the scene is provided via a small window whereby non-HMD users can observe their relative distance from the HMD users in the virtual space, which improves their assessment of distance (as shown in Figure 4). In addition, when non-HMD users remotely engage in the VE with HMD users, confirming their position in the VE through a third-person viewpoint helps improve their awareness and understanding of the activities of other users in the VE, thereby increasing immersion and co-presence. In addition, we added a text-based UI to alert the user if any other user is close by (as shown on the left side of Figure 4). The distance between the users is calculated from the position coordinates (x, z-axes) of the VE user. If the distance is close to the threshold value, the text information provides hints to users.

### 3.3. Implemented Experiences

The main goal of the proposed low-asymmetry interface was to enable non-HMD and HMD users to feel a similar level of presence and enjoyment. To verify the effectiveness of the proposed system, we implemented two applications (maze, racing game), and based on the two applications, three experiments were conducted to investigate our system.

For the first experiment, we designed a maze game to test the main functions based on a walking task to compare the non-HMD users’ immersion and the system usability when moving with the single walking simulator and touch input in a low-asymmetry environment. For the second and third experiments, we designed a running race game for a comparative study with all users. The second experiment analyzed the HMD and non-HMD users’ experience of presence in a shared environment with the proposed low-asymmetry interface and basic interface (the details are described in Section 4.3). The third experiment compared the enjoyment between HMD user and non-HMD users, also compared the all users’ enjoyment between one-to-one and one-to-N environments. A racing game was especially chosen because in most multiuser collaborative applications, the user relationships at the same experience level are either competitive or cooperative. We designed the racing game scenario in two forms, i.e., “general running” and “relay running”, representing the competitive and cooperative cases, respectively, for HMD and non-HMD users, and both types of users played equally influential roles (the details of the experiments are presented in Section 4.3 and Section 4.4).

## 4. Experiments and Analysis of Results

### 4.1. Environments and Participants

The application and environment developed for the HMD and non-HMD users based on a low-asymmetry system were implemented using Unity 3D 2019.2.17f1, Oculus SDK, and a walking simulator used for user walking motion determination, as presented by [8]. The functionality of the walking simulator was implemented using an Arduino Sketch 1.8.12. The network implemented for the HMD and non-HMD users to join the same virtual world was based on photon unity networking. The mobile-device system for non-HMD users was based on Android 4.3. The PC configuration used for the system design and experiments was Intel Core i7-6700, 16 GB RAM, and a GeForce 1080 graphics card.

**Figure 5:**
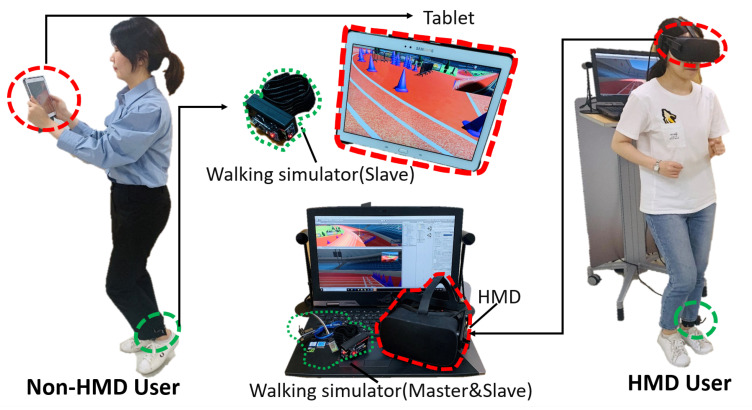
Low-asymmetry system ((Left) Green part: Devices for non-HMD users, including a tablet for visualization and a walking simulator connected to the tablet though Bluetooth. (Right) Red part: HMD gear and walking simulator for the HMD users).

We conducted experiments in a 7 m × 7 m room, because in our system the non-HMD user devices are wireless and can move freely, and therefore, we did not restrict the location of the non-HMD users. Because the VR application of our system involved movement determination, the users were standing up during the experiments (as shown in Figure 5).

To achieve a systematic analysis of the proposed system, we surveyed 12 participants (5 female and 7 male), who were 19 to 35 years old, with previous VR experience and an average age of 24.3. Each participant engaged in all the experiments. In the first experiment, the participants were tested independently. In the second experiment, we divided the participants into six pairs. In the third experiment, we divided the participants into four groups of three participants.

### 4.2. Immersion of Non-HMD Users

**Figure 6:**
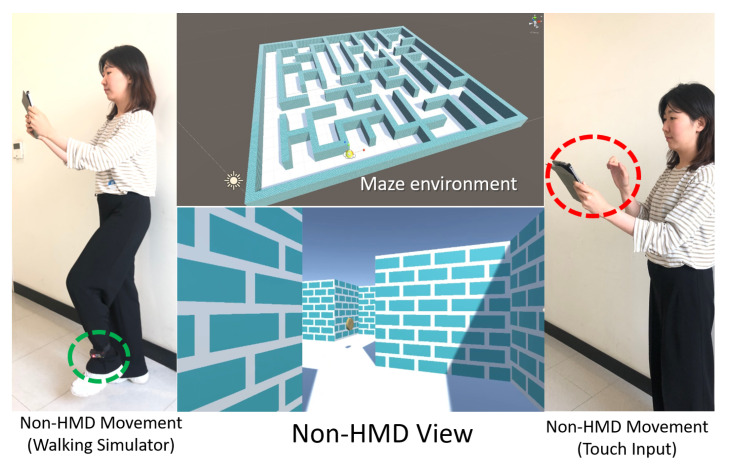
Experimental environment for non-HMD user movement using a walking simulator and touch input.

Experiment: The first experiment aimed to evaluate the immersive properties and usability of the system for non-HMD users while they were moving with the walking simulator and performing touch inputs in a mobile environment. Previous researchers [8] analyzed the walking realism for HMD users when using a walking simulator and keyboard, concluding that moving via a walking simulator in a maze environment is more realistic and immersive than moving via keyboard actions. In our experiment, we also conducted the experiment in a maze environment, but we focused on non-HMD users performing the same task of moving in a maze to reach all coins (as shown in Figure 6).

User Study: For this study, we recruited 12 participants to compare movement using the walking simulator with that via the touch input. To evaluate the experience provided by the walking simulator, in the first survey we asked the three questions referenced by [8,44] to analyze the immersive quality for non-HMD users. The Likert scales ranged from 1 (not at all) to 5 (extremely) underlying continuous measures and assigned equally spaced scores to response categories.

Q1. How easy was it to control movement in the VE?
Q2. Was the movement accurately controlled in the VE?
Q3. Did you feel that the VE provided an immersive experience?

**Figure 7:**
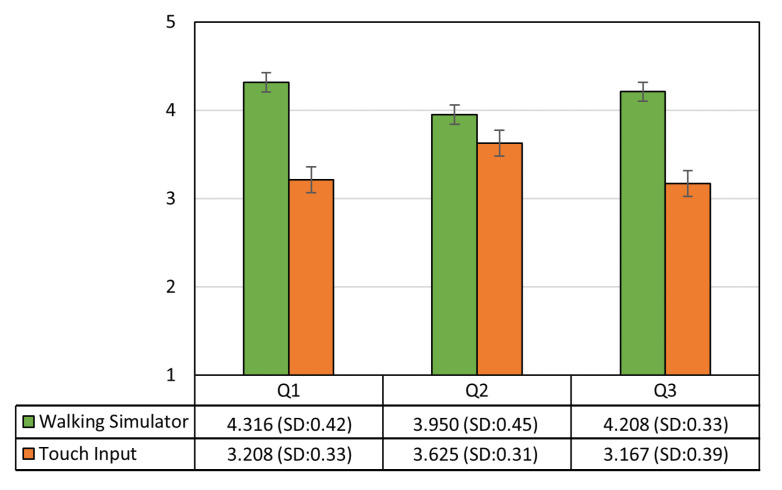
Survey results of the immersive experience of non-HMD users while moving with the walking simulator or touch input.

Result Analysis: The first question checked the convenience and intuitiveness experienced by the user during movement when using the provided mobile device. According to the results shown in Figure 7, the mean values for walking simulator-based and touch input-based movements were 4.316 (SD = 0.42) and 3.208 (SD = 0.33), respectively. The scores were analyzed using the independent *t*-test, showing that the score for walking simulator-based movement was significantly higher than that for touch input-based movement (t(22)=6.178,p<0.001). This shows that mobile control using a walking action is more convenient and intuitive than moving by using manual touch operations on a mobile device. The second question was regarding the accurate control of movement in the VE, and the results showed that the mean values for both movement control methods were similar, that is, both methods controlled the movement accurately (see Figure 7). The third question was regarding the immersive experience of the users, and the results showed that the mean value for walking simulator-based movement is higher than that for touch input-based movement (t(22)=7.033,p<0.001). The scores were 4.208 (SD = 0.33) and 3.167 (SD = 0.39), respectively.

**Figure 8:**
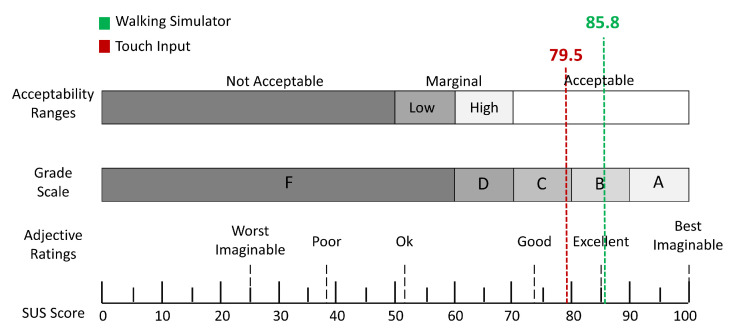
SUS scores for walking simulator-based movement and touch input-based movement [45].

In addition to the above analysis, we conducted a user study using the system usability scale (SUS) [45] to verify the effectiveness of the single-leg-based walking decision method and compared the two movement-control methods. The study included a 10-item Likert 5-point scale (1 to 5 points). The average SUS score was 68, according to the results shown in Figure 8, the scores for walking simulator-based movement and touch input-based movement were 85.8 and 79.5, respectively. We can conclude that although both movement types achieve an above-average score, the single-leg-based walking decision method is useable and reasonable, and the usability of the walking simulator is higher than that of the touch input. The survey results confirmed that walking simulator-based movement has a higher satisfaction for non-HMD users, although the experience is comparable to touch input-based movement.

### 4.3. Presence

**Figure 9:**
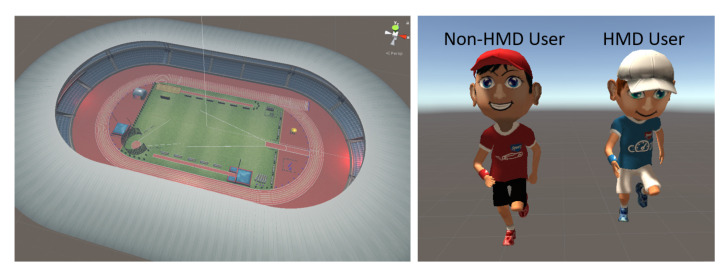
Environment and characters of the racing game experiments.

**Figure 10:**
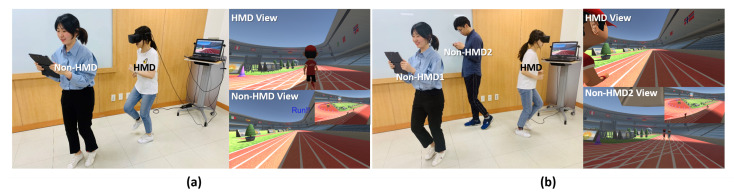
(**a**) One HMD and one non-HMD user can play in the relay running mode. (**b**) One HMD and more than one non-HMD user can play in the general 100 m race mode.

Experiment: This experiment analyzed the presence experienced by HMD and non-HMD users in the proposed low-asymmetry and the basic interface systems. We applied the two systems to a VR environment representing a simple running scenario (as shown in Figure 9), where both HMD and non-HMD users play equally influential roles. In these experiments, the relay running mode requires the users to be involved in a cooperative relationship. The HMD and non-HMD users stand at different starting positions in the same lane to enable relay running. We set the total distance for the relay run as 100 m (distance measured by x-axis coordinate); further, the initial position of the non-HMD user is at the start position of the race, whereas that of the HMD user is 50 m away from the non-HMD user’s initial position. In the relay running mode, we used a text-based UI to alert the user to start running (as shown in Figure 10a).

As mentioned before, we investigated the presence experienced by both HMD and non-HMD users with equally significant roles in the low-asymmetry interface system and the basic interface system. For movement control, the basic interface provides a controller to the HMD user and a mobile tablet to non-HMD users, which they control according to touch input.

User Study: We used the presence questionnaire [44], which includes questions regarding realism, possibility of action, quality of interface, possibility of examination, and self-evaluation of performance, to analyze the HMD and non-HMD users’ presence in the shared environment. For this study, we recruited 12 participants in pairs (one HMD and one non-HMD user), with each of them having experience with both wearing and not wearing the HMD.

**Figure 11:**
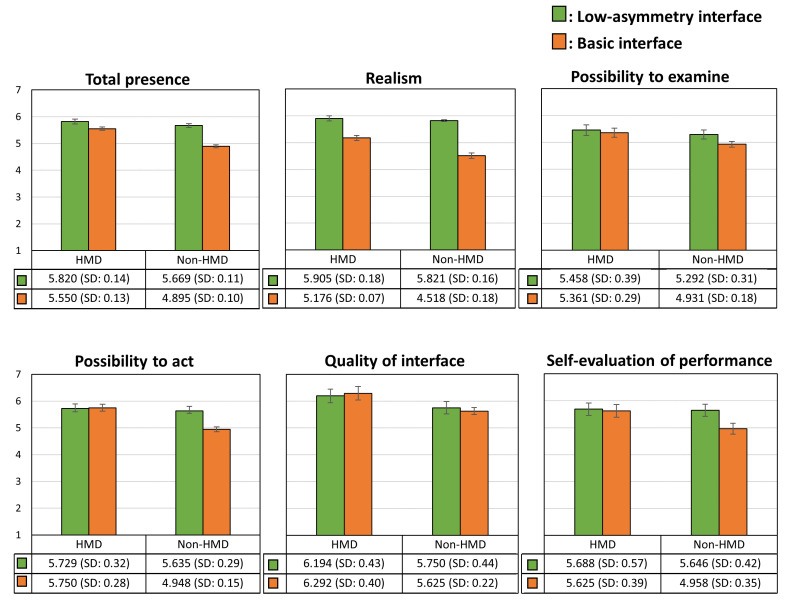
Presence questionnaire results for HMD and non-HMD users.

Result Analysis: Figure 11 shows the results of the presence questionnaire that examined the experiences of HMD and non-HMD users in the two interface systems. According to the results, we found that the difference in the total presence experienced by the non-HMD and HMD users for the low-asymmetry interface (t(22)=2.701,p=0.013) was not statistically significant; however, in the basic interface (t(22)=14.19,p<0.01), the experienced presence was significantly different.

Notably, the realism experienced by the non-HMD users (t(22)=18.74,p<0.01 ) in the low-asymmetry interface (M=5.821,SD=0.16) is significantly higher than in the basic interface (M=4.518,SD=0.18). The term realism signifies whether it is possible to control the movement naturally and the VE experience is comparable with the real-world experience. Realism in controlling movement is especially important because the walking simulator detects real actions to ensure that the user’s movement in the VE is controlled by actual leg movements. Therefore, it can reduce the sense of inconsistency between the VE and the real world. Simultaneously, visual and perspective information is also an important factor affecting realism. In previous studies [5,46], it was shown that using both first- and third-person viewpoints can significantly improve the realism and presence experienced by non-HMD users. In our system, we ensured that in addition to a first-person viewpoint, non-HMD users were also provided with a third-person viewpoint, enabling them to grasp their own position in the VE and the distance from HMD users. Thus, the interference of the physical environment with the VR vision can be reduced for non-HMD users, and the experienced realism and presence can be enhanced.

Finally, for the question “How much delay did you experience between your actions and expected outcomes” in the questionnaire, we found that the low-asymmetry interface does experience a latency problem compared with the basic interface because of the time taken for transferring the walking simulator data over a network, and the corresponding difference is significant for the non-HMD users (t(22)=3.675,p<0.001).

### 4.4. Enjoyment

Experiment: In the final experiment, we not only experimented with the case of a one-to-one relationship between an HMD user and a non-HMD user but also considered the one-to-many case of one HMD user and multiple non-HMD users in the shared VE. We analyzed the enjoyment of the users of our system via the running application, which is different from the relay running mode described in Section 4.3. In this experiment, we analyzed the user experiences with regard to the general 100 meter race mode. Here, the users’ relationship is competitive, and the users will be running from the same starting position but in different lanes.

User Study: For this study, we analyzed the users’ enjoyment by evaluating their positive feelings according to their responses to the post-game module (the questions related to the positive experience) of the game experience questionnaire (GEQ) [47]. We divided the 12 participants into four teams, each with one HMD user and two non-HMD users (see Figure 10b). Each team participated in the race game three times, with each user in a team playing the non-HMD role twice and the HMD role once.

**Figure 12:**
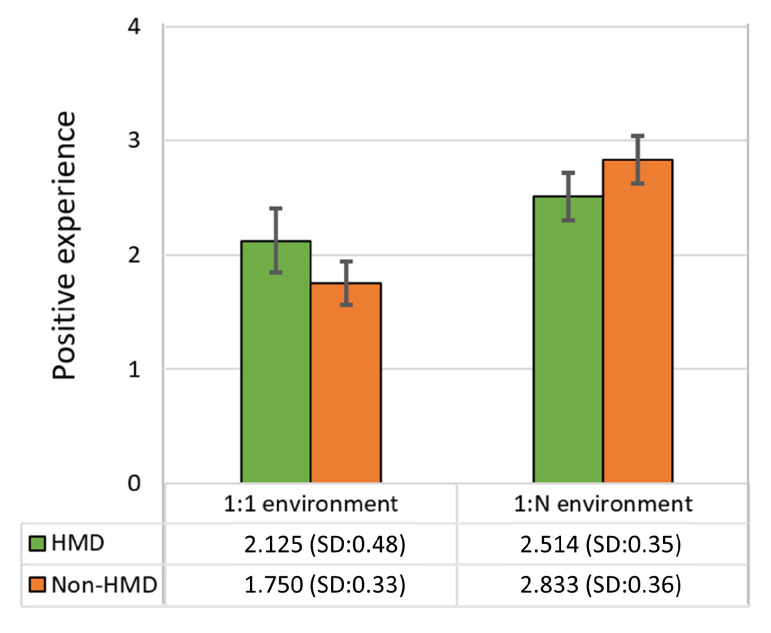
Mean (SD) of the results for the post-game module of the experience questionnaire (GEQ). According to the results for positive experiences, non-HMD players in a one-to-many environment reported higher scores than those playing in a one-to-one environment.

Result Analysis: Figure 12 illustrates the results for enjoyment when HMD and non-HMD participants played in the one-to-one and one-to-two environments. As shown on the Figure 12, the difference in the positive experience of non-HMD users between the one-to-one and one-to-N environments is significant (t(22)=7.661,p<0.01), however, the difference of HMD user between one-to-one and one-to-N environments is not statistically significant (t(22)=2.58,p=0.011). Furthermore, in the one-to-one and one-to-N environments, the difference between HMD and non-HMD users is not significant (t(22)=2.22,p=0.018;t(22)=2.17,p=0.02), respectively.

We can conclude that in the one-to-one and one-to-N environments the enjoyment of non-HMD users is similar to the HMD user and the non-HMD user’s enjoyment increased in the one-to-N environment. When the multiple non-HMD users engaged in the VE, the score as both “I felt satisfied” and “I felt proud” in the questionnaire is significantly higher than when one non-HMD user engaged (t(22)=3.334,p<0.01;t(22)=3.490,p<0.01), respectively. Because in this competitive running game content, the participation of multiple users increased competitiveness, and since the non-HMD users can confirm the other participants’ position in VE at any time through the third-person viewpoint and observe the real physical reaction when users are co-located, the one-to-N cases enable to enhance the non-HMD users’ experience more than one-to-one cases.

However, the enjoyment of the HMD user is not increased significantly. The previous work [48] have proved that to design optimized roles for HMD and non-HMD users can increase their enjoyments. According to the previous work [48], we analyzed one of the reasons for the result is that the experiment content and the role of users are designed relatively simple. In this experiment, all users are assigned the same role and as mentioned before, the third-person viewpoint enables the non-HMD users to confirm the participates’ position and number at any time in VE, while in the case of HMD user, if other participants’ position is behind of HMD user, the HMD user is difficult to perceive the number and position of other participants. The HMD user is affected by the content and the single role to a degree so that the difference was not significant between one-to-one and one-to-many cases in this experiment.

### 4.5. Discussion

In this study, the main goal was to examine the effect of a low-asymmetry interface on the presence and experience of all users in a shared environment. First, we conducted an analysis of the proposed single-leg-based walking decision method and found that its usability in the walking-detection task, as well as the users’ immersion while using physical navigation was significantly higher than that of virtual navigation (touch-input-based movement). In addition, we compared the proposed interface with the basic interface to analyze the experience of presence. The result shows that non-HMD users experience a presence similar to that of the HMD users in our low-asymmetry interface. In terms of realism, there is a significant difference between the proposed interface and the basic interface. In addition, we compared the one-to-one and one-to-two engagement cases to analyze the experience of enjoyment. According to the results, we found that the experience between HMD and non-HMD users were similar in the both environments. In the one-to-many environment, as more participants enriched the content, the enjoyment of non-HMD users was significantly more positive than that in the one-to-one environment.

These findings confirm that a low-asymmetry interface can increase the presence of non-HMD users such that it is comparable to that of HMD users. In addition, these findings demonstrate that in the proposed system, the greater the participation of non-HMD users, the more enhanced is the enjoyment of non-HMD users. Furthermore, because the non-HMD users’ enjoyment is similar to the HMD user, the finding confirms the low-asymmetry interface can provide immersion to non-HMD users in the VE.

When compared with existing approaches [1], our system enables open participation. Because the device used by non-HMD users in our system has no space restrictions, they can either be co-located or remote, enabling a shared VR experience with HMD users in one-to-many environments. A non-HMD device impacts the field of vision of the non-HMD users and decreases their immersion; however, by using a portable mobile device, non-HMD users can positively perform physical interactions (e.g., physical touch) with HMD users in a co-located shared VR experience, the feeling of presence can be improved, and the non-HMD users’ enjoyment can be increased. Although only simple scenarios were examined in our experiment, the locomotion-based shared experience achieved in our system can be subsequently applied to a wide variety of applications, such as a competitive game of PacMan or a running game, or to cooperative tasks such as a VR maze and relay running. Here, non-HMD users can contribute independently or collaboratively with HMD users in the shared environment.

## 5. Conclusions

In this paper, we propose a low-asymmetry system that can improve the immersion and presence experienced by non-HMD users in a VR environment shared with HMD users. Our proposal includes providing a portable mobile device for non-HMD users to ensure that they have their own first-person viewpoint and a walking simulator for all users to ensure movement realism. Because the proposed walking module supports in-place movement, even a small physical experimental space can allow wide-ranging movement in the VE. Further, as we are able to utilize single-leg-based walking measurements, the walking simulator itself is more convenient and simpler than those used in most existing systems. Using a portable mobile device and a walking simulator can offer non-HMD users similar perspectives and movement capabilities as those available to HMD users, enabling non-HMD users to play the same roles as HMD users in an equally rewarding shared environment. Because the devices used in our system for non-HMD users are low-cost and portable, our system supports simultaneous participation by multiple non-HMD users. To investigate the HMD and non-HMD user experiences of the proposed system with respect to presence and immersion, we created a simple running application in a low-asymmetry VR environment in which the main interaction is the users’ movement. According to the experimental results, the non-HMD users reported similar levels of immersion and presence to those experienced by the HMD users.

One of the limitations of our current system is that it is restricted to a single HMD user to avoid the chance of a physical collision between users in a co-located environment. In addition, immersive interaction via hands was not considered. We aim to address the hand interaction issue in future work by incorporating a hand-tracking sensor similar to Leap Motion controller and a system for hand gripping [49]. In addition, the third-person viewpoint with a fixed position, which was used in this study, cannot be applied to large-scale scenes. In future work, we will improve the third-person viewpoint to a movable view suitable for a large-scale scene. In addition, we aim to improve the walking simulator for determining more varied motions. Finally, because the proposed low-asymmetry interface can be adopted in a wide variety of basic movement interactions, we will apply it to various popular VR applications to improve the quality of our experimental results.
